# Aptamers as Both Drugs and Drug-Carriers

**DOI:** 10.1155/2014/697923

**Published:** 2014-09-11

**Authors:** Md. Ashrafuzzaman

**Affiliations:** Department of Biochemistry, College of Science, King Saud University, Riyadh 11451, Saudi Arabia

## Abstract

Aptamers are short nucleic acid oligos. They may serve as both drugs and drug-carriers. Their use as diagnostic tools is also evident. They can be generated using various experimental, theoretical, and computational techniques. The systematic evolution of ligands by exponential enrichment which uses iterative screening of nucleic acid libraries is a popular experimental technique. Theory inspired methodology entropy-based seed-and-grow strategy that designs aptamer templates to bind specifically to targets is another one. Aptamers are predicted to be highly useful in producing general drugs and theranostic drugs occasionally for certain diseases like cancer, Alzheimer's disease, and so on. They bind to various targets like lipids, nucleic acids, proteins, small organic compounds, and even entire organisms. Aptamers may also serve as drug-carriers or nanoparticles helping drugs to get released in specific target regions. Due to better target specific physical binding properties aptamers cause less off-target toxicity effects. Therefore, search for aptamer based drugs, drug-carriers, and even diagnostic tools is expanding fast. The biophysical properties in relation to the target specific binding phenomena of aptamers, energetics behind the aptamer transport of drugs, and the consequent biological implications will be discussed. This review will open up avenues leading to novel drug discovery and drug delivery.

## 1. Introduction

The search for new molecules to be used as drugs or drug carriers increased within the last decade. Cellular structure and function disorder based diseases require increased attention. Cancer, Alzheimer's, and neurodegenerative diseases are especially mentionable among the diseases under scrutiny. Other types of common diseases like infectious diseases and obesity require fast attention too. Drug discovery for these various classes of diseases falls within urgent needs. Repair of most of the biological structure disorders requires drugs to reach directly the disorder sites. Then the drugs act upon the specific structures responsible for causing respective diseases. Many of these structures are proteins, microtubules, lipids, nucleic acids, and similar large or small biomolecules. Chemical intervention is a prime choice in doing so (see a few cases in [1–3]). For such intervention discovery of target specific chemicals or molecules needs to be made. These agents would reach easily the target structures. The whole process can be divided into two categorical action phases, namely, drug molecules being released to the target sites and molecules showing target specific interactions resulting in some sort of physical binding. To gain clarity one can read a few case studies performed using various biophysical techniques as mentioned here. The understanding of chemotherapy drugs' bioavailability at the biological structures responsible for causing cancer [4–6], the development of the aptamer designing formalisms for ensuring target molecule specific binding of agents [7–11], and addressing the ion channels' stability inside lipid membranes through electrical charge based interaction energetic coupling [12] are among a few best ones found so far. The computational scientists like computational chemists use computer aided drug design to make drug library. The members of any such library are chosen based on mainly the drug-target binding energy calculations. Various* in silico* techniques like molecular dynamic (MD) simulations, quantum mechanical (QM) calculations, and docking (see [2]) primarily detect candidate molecules for general binding to specific targets. These techniques fail to provide enough information on binding phenomena and energetics, though they may be utilized to address the distance dependent energy values as a result of molecular level physical interactions [13]. Further* in vitro* experiments validate the physical binding potency and thus drug molecules usually are ranked based on binding stability and energetics [13]. There have been other traditional cell based dose-response techniques historically used. Here any disease is tried with the doses of various chemicals and the drug efficacy is investigated. This traditional biochemistry based drug discovery technique lacks heavily to address substantial molecular level understanding of drug-target binding. They cannot often distinguish between target and off-target specific actions. Lack of enough candidate chemicals also makes the traditional techniques not substantial enough to independently tackle the increasing demands in drug discovery sciences. But the biochemical techniques are still applied on candidate drug molecules to finalize the optimal choices on the drug candidates creating a drug bank for specific target structure. This way drug banks for various diseases are primarily created. Through modification in structural and physical properties of any drug molecule's active structural sites many new candidates in the same category can be created. The number of the members in a group thus increases and the candidates appear with different quantitative binding potencies towards specific targets [2, 14]. Among various chemicals colchicine [2, 14], taxol [15–18], aptamers [7–11], and so forth are especially mentionable that are used for modifications and thus to create drug banks. The primary targets of these molecules are different or we can consider these molecules to be target specific. But occasionally they are found to show substantial off-target binding that accounts mainly for “drug cytotoxicity.” Due to cytotoxicity properties some molecules also show important off-target fractional binding specificity [13]. Thus same drug may appear as useful for other purposes. In this review, I shall focus on understanding aptamer's [19, 20] efficacy for various medical purposes. I shall specifically analyze how aptamers [19, 20] (i) can be designed using various techniques, (ii) can be used as drugs to target certain structural sites that are responsible for various diseases, and (iii) can be used as drug carriers to carry other drug molecules to cell based target structural sites. I shall also address aptamers' off-target effects and consequent cytotoxicity, in general.

Several unique properties of aptamers [19, 20] make them attractive tools for use in a wide array of molecular biology applications and, especially, as potential pharmaceutical agents. First, most aptamers bind to targets with high affinity, demonstrating typical dissociation constants in the pico to nanomolar range. Binding sites for aptamers include clefts and grooves of target molecules (including enzymes) resulting in antagonistic activity very similar to many currently available pharmaceutical agents. Second, aptamers are structurally stable across a wide range of temperature and storage conditions, maintaining the ability to form their unique tertiary structures. Third, aptamers can be chemically synthesized, in contrast to the expensive and work-intensive biological systems needed to produce monoclonal antibodies. Fourth, as aptamers are mainly nucleic acid based compounds their biological adaptation to targets would perhaps be with moderate or no toxicity. All these general properties make aptamer tools relatively special in drug discovery science.

I shall now address a brief background on aptamer discovery [19, 20] and subsequent developments. In 1989 Tuerk and Gold discovered in a chemistry laboratory in University of Colorado what could be practically used as an alternative to antibodies [19]. The duo thus emerged as discoverer of systematic evolution of ligands by exponential enrichment (SELEX) and the iterative* in vitro* process for selecting and amplifying RNA molecules to bind with specific molecular targets. Almost simultaneously, Harvard University researchers Ellington and Szostak published a paper entitled “*In Vitro* Selection of RNA Molecules That Bind Specific Ligands” in journal Nature where they termed the RNA oligos/molecules as “aptamers” [20]. Since these aptamer discovery scientists have been engaged to find target specific drugs using nucleic acids RNA and DNA oligos, aptamers are expected to be doing same kind of things as well as antibodies but at a cheaper cost and a lesser toxicity. In drug discovery, the search for aptamers as candidate molecules is undoubtedly expected to rise in coming years. So far some visible progresses have already been made [21].  Table 1 summarizes a group of aptamers that are in various stages of drug discovery.

Table 1 clearly shows that aptamers are in process of invading the drug discovery avenues to appear as drugs for different kinds of targets. They are now tried as medicines or possible candidates for treating age-related macular degeneration [22–24], cancer [11, 25–28], acute coronary syndrome [29, 30], thrombotic thrombocytopenic purpura [31], coronary artery bypass grafting [32], diabetic nephropathy [27, 33], hemophilia, anemia [34, 35], and so forth. It has already covered a wide range of interests. I shall provide an in-depth analysis on how the aptamers are designed from both RNA and DNA oligos. I shall then focus on addressing the possible applications currently under considerations and some general properties regarding their use as drugs and drug carriers.

## 2. Aptamer Designing Templates

Aptamer designing techniques depend on the mechanisms of how the aptamers interact or bind with their targets. Aptamers are three-dimension structure forming therapeutic oligonucleotides. Their structures and related physical properties like geometry and charge residues are expected to dictate in the target interaction raised creation of stable or semistable complex structures in the vicinity of the binding regions. In binding with targets aptamers show high affinity and specificity [11]. Unlike small interfering RNAs (siRNAs), antisense oligonucleotides, and so forth, that inhibit protein translation by Watson-Crick base-pairing to their respective messenger RNAs, aptamers bind to targets in proteins and occasionally to other targets, for example, lipids [11]. In this section, I shall discuss various techniques that have so far been used to design aptamers targeting various binding sites. Some of them are considered to be general techniques while others as specific for specific targets. All the major techniques theory, experiment, and computation are greatly in use to design aptamers. They will be detailed here.

### 2.1. SELEX

**Figure 1:**
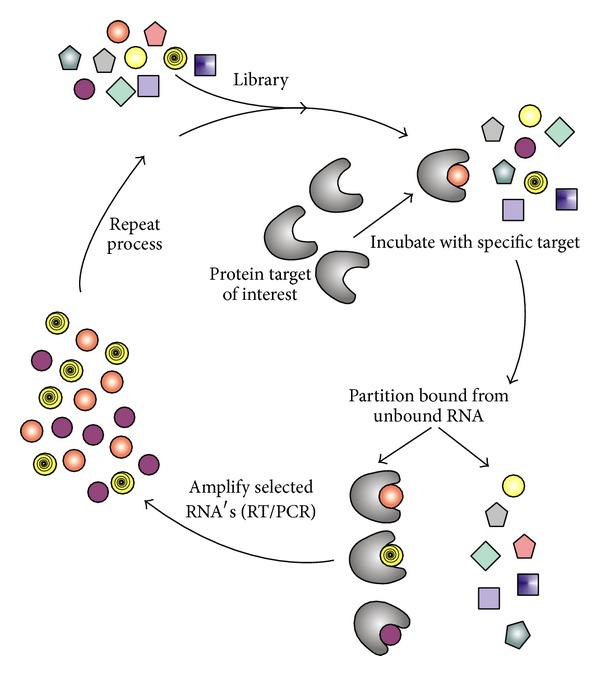
Schematic of SELEX. This figure is taken with due permission from [36].

In designing right aptamers for interactions with binding sites in specific targets an iterative screening process termed SELEX on complex nucleic acid libraries has been used since its discovery more than two decades ago [19, 20]. The SELEX consists of iterative rounds of affinity, purification, and amplification. Over successive rounds, the pool becomes enriched with ligands that bind the target protein with high affinity and specificity. For a developed schematic diagram see Figure 1 [36]. SELEX was developed for designing a randomized RNA/DNA library of appropriate complexity and aptamer length to provide candidates for binding with specific targets [19, 20]. For general background of SELEX and aptamers, readers are encouraged to read the two breakthrough making discovery papers [19, 20] and a few subsequent reviews [7–10, 37].

As discussed above use of SELEX helps in selecting short RNA/DNA sequences that would bind to specific biomolecular targets. These targets include small molecules, proteins, nucleic acids, phospholipids, and complex structures like cells, tissues, bacteria, and other organisms. The selected aptamers have strong and specific binding properties through molecular recognition. They are promising tools in studying molecular biology with recognized therapeutic and diagnostic clinical applications [7–10, 37]. SELEX consists of a number of rounds of* in vitro* selection in which the RNA/DNA pool is incubated with the binding target. The nonbinding or loosely binding sequences are discarded, while the binding sequences are expanded using the polymerase chain reaction method to provide a pool of sequences for the next round of testing. In practice, multiple rounds of selection and expansion are required before unique tightly binding sequences can be identified. Additionally, isolated aptamers will often need to be reengineered to reduce their sequence length and impart additional favorable biological properties. These issues pose a challenge for the efficient identification of correct aptamers [11, 38]. Although SELEX has been proven to be strong technique for target structure specific selection of small oligos it does not guarantee to be so simple. Search for simpler techniques therefore continues. Using theoretical, computational and experimental methods a few alternatives have already been proposed [11, 39–42] including one by our group [11].

Over the past several years, various research groups have attempted to develop theoretical methods to speed up the SELEX process and to design optimal aptamer sequences. Despite theoretical modeling of the working principle involved in SELEX [39], various* in silico* approaches are favored by many groups [40–42]. There are two general directions pursued so far in this area: (i) one with a focus on developing a computational design of structured random pools such as RagPools [40, 41] and (ii) an application of a virtual screening process [42]. Our group has recently proposed an entropic fragment based approach (EFBA) which combines computational technique and experimental validation to finally select right aptamer candidates for target specific binding [11]. I shall provide brief descriptions on all these three additional techniques that were developed during post-SELECX discovery era.

### 2.2. Computational Design of Structured Random Pools

**Figure 2:**
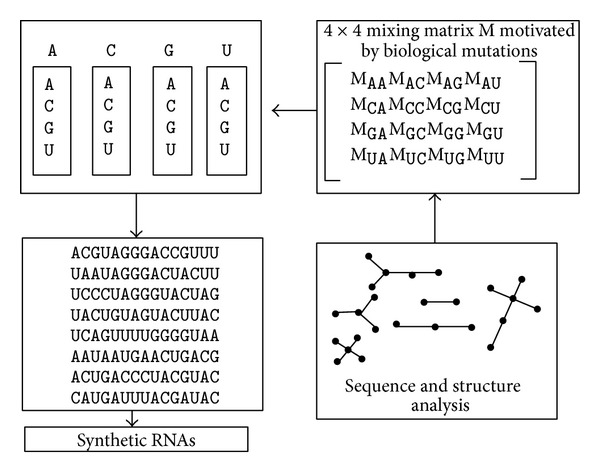
Modeling the RNA pool generation process using mixing matrices and analysis of pool structural distributions using tree graphs. This has been redrawn in light of figure presented in [41]. The mixing matrix applied to any starting sequence specifies the mutation rates for all nucleotide bases. The matrix elements of each row represent nucleotide base (A, C, G, and U) composition in a vialor synthesis port. Mixing matrices and starting sequences can be optimized to yield target structured pools.

Since the discovery of SELEX [19, 20] the* in vitro* selection technology has been used. But it is found to be a cumbersome iterative experimental process. Computational methods may help initiate the selection process. Kim et al. [40, 41] have recently proposed a computational technique for designing structured RNA pools for* in vitro* selection of RNAs. Here the main aim is to enrich the* in vitro* selection pools with complex structures and thus increase the probability of discovering novel RNA molecules. This group [40, 41] has recently developed an approach for engineering sequence pools that links RNA sequence space regions with corresponding structural distributions via a ‘‘mixing matrix” approach combined with a graph theory analysis. Here five classes of mixing matrices have been defined motivated by covariance mutations in RNA. The constructs define nucleotide transition rates and they are applied to choose starting sequences to yield specific nonrandom pools.  Figure 2 [41] illustrates the relations among pool sequence/structure analysis, mixing matrix and starting sequence, and pool synthesis.

In contrast to random sequences, which are associated only with a local region of sequence space, this computational technique helps design pools that can target specific motifs.

Kim et al. developed computational RNA pool design algorithm that involves five simple steps [41]. They are described here. (i) First a target distribution of topologies or shapes is specified. (ii) Then candidates for starting sequence and mixing matrices based on covariance mutations are defined. (iii) Shape frequency distributions corresponding to all starting sequence/mixing matrix pairs are computed (for details see [54]). This step analyzes pool structural diversity. (iv) To approximate the designed pool the number of mixing matrices is chosen. (v) And finally, a target structural distribution by optimizing a set of starting sequence/mixing matrix pairs based on pool structural frequency data is approximated. The experimental synthesis of designed pools can benefit from using optimized starting sequences, mixing matrices, and pool fractions associated with each of the constructed pools as a guide.

To let experimentalists use the design approach the same group [41] has developed a companion web server, RAGPOOLS (RNA-As-Graph-Pools), which is used for designing and analyzing structured pools for* in vitro* selection. RAGPOOLS aims to help design structured RNA pools with target motif distribution, analyze structural distributions of RNA pools, and stimulate discoveries of novel RNAs via combined experimental and theoretical pool design [41].

### 2.3. Virtual Screening Process

After the appreciable discovery of a computational approach to develop RNA pool by Kim et al. (discussed previously) a virtual screening approach was proposed by Chushak and Stone [42]. This is a computational approach for designing a starting pool of RNA sequences for the selection of RNA aptamers for specific analyte binding. Here three steps are followed: (i) RNA sequences are selected based on their secondary structure. (ii) A library of three-dimensional (3D) structures of RNA molecules is created. (iii) We then fix a small desired molecule to which our aptamer needs to bind. A high-throughput virtual screening of the created library is used to select aptamers with binding affinity to the specific small molecule.

**Figure 3:**
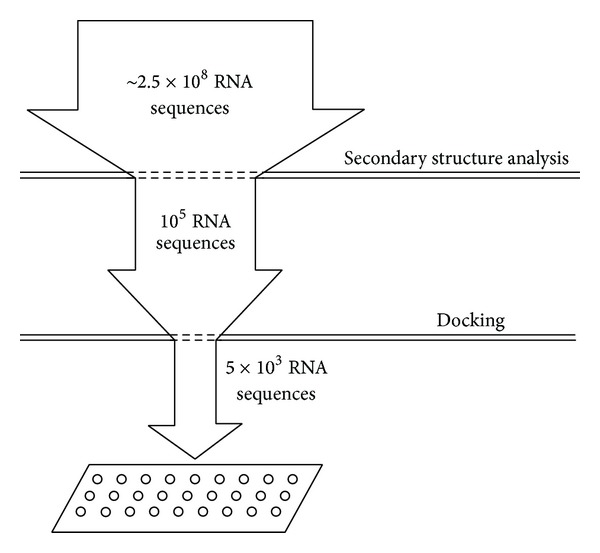
Reduction in size of the RNA sequence space for experimental screening and selection of RNA aptamers by* in silico* approach. The secondary structure of more than 2.5 × 10^8^ RNA sequences was analyzed to select 10^5^ sequences for the RNA 3D structure library. The high-throughput virtual screening of the developed library selected 10^3^-10^4^ sequences suitable for the experimental screening and verification.

The* in silico* approach to create a list of RNA sequences with potential binding affinity to a desired small molecule is developed on three simple steps (see Figure 3, taken with due permission from [42]).

- Selection of RNA sequences based on their secondary structure: the free energies in the secondary RNA structure formation is found significantly lower than the same-length random sequences [55]. These structural motifs maximize the presence of stable low-energy structures. This criteria selection screens one RNA sequence from about 2500 random sequences.
- In step 2, the 3D structures of RNA are computationally predicted using available computational techniques and programs. Use of simplified energy function that takes into account the backbone conformational and side-chain interaction preferences observed in the experimental RNA structures [56], AMBER force field [57], and generalized Born implicit solvent [58] helps generalize the technique. For conformational flexibility, various lowest energy structures for each sequence are placed into a library of RNA molecules to perform ensemble docking.
- In step 3, the library of RNA molecules is screened. Computational docking is usually used to identify small-molecule ligands that bind to proteins [59].

The above-mentioned proposed approaches reduce the RNA sequences search space by four to five orders of magnitude. Thus it accelerates the experimental screening and selection of high-affinity aptamers.

Both general directions explained in previous Sections 2.2 and 2.3 possess some disadvantages. The first one (Section 2.2) is hindered by the requirement of a library of reasonably good starting sequences. It also requires prediction tools to arrive at candidate structures and the associated structural distributions. While this direction provides optimal choices for the design of a random pool for SELEX-prepared structures, one still needs to go through the SELEX process to identify the best aptamer in the final aptamer selection. The second direction (Section 2.3) requires structural information about the binding interfaces, reasonable prediction of the tertiary structures of nucleotide sequences, and the availability of immense computational resources. One has to screen at most 4^N^ N-mer RNA/DNA sequences to be able to analyze sequence diversity and structural complexity. The required computational power would be prohibitively large to complete the screening process. Another challenge involves the prediction of tertiary structures for arbitrary RNA/DNA sequences. Although the development of the RNA/DNA 2D and 3D structural prediction methods is promising, their validation is still questionable. We have developed a novel technique [11] which designs aptamer candidates directly based on knowledge of the target structures. Here theoretical and computational techniques are used to design the molecules. We have also proposed a valid experimental technique for a test/example case. The next Section 2.4 will give details about our technique.

### 2.4. Entropic Fragment Based Approach (EFBA)

As discussed earlier SELEX requires designing randomized RNA/DNA libraries of appropriate complexity and aptamer length to provide candidates for binding with the specific targets. These are laborious and time-intensive procedures. To overcome some of the problems our group has proposed an alternative technique to design aptamers. In this section, I shall mainly focus at our group developed novel aptamer discovery strategy that covers techniques like theoretical,* in silico*, and* in vitro* assays. We have proposed an information-driven theoretical approach which is an entropy fragment-based approach (EFBA), for the computational design of aptamer templates based solely on structural information of the biomolecular targets [11]. This approach is free from some of the impediments of SELEX as mentioned above. Regarding aptamer design the detailed method of maximum entropy is described in [60–68]. I wish to provide here brief description on our novel technique applied for aptamer design (for details see [11, 38]).

The most important feature of information-driven theoretical approach is that it designs aptamer templates based solely on the structural information about biomolecular targets. Therefore, as new information on the target structure becomes available we can engineer these templates either theoretically or experimentally for further optimization. This ensures better binding affinity without going through the SELEX reiterative processes. The key to this approach lies in answering the question: “given the structural information of the target, what is the preferred probability distribution of having an aptamer that is most likely to interact with the target?” We have proposed integrating the concept of information processing with the seed-and-grow strategy to answer this question and design templates for aptamers. Three methods regarding information processing are considered: (i) a method of assigning probability distributions based on a limited amount of information denoted by MaxEnt [60, 61]; (ii) a method of updating probability distributions from a priori when new information becomes available denoted by ME [62, 63]; and (iii) a selection criterion for different probability distributions associated with various types of nucleotides [65–68].

To test the hypothesis described here we considered to address the aptamer designing mechanisms for two targets: (i) protein thrombin (with 232 amino acids), a 37-kDa coagulation protein (PDB ID: 1HUT), which has an identified aptamer binding site [69], and a binding aptamer which was previously determined through SELEX [70], and (ii) phospholipid phosphatidylserine (PS) because of our interest in apoptosis, where externalization of PS is an early indication that this process has been initiated in the cell [71]. As this is pretty new I shall address some details about the designing techniques, experimental validation, and possible applications here.

#### 2.4.1. Theoretical Analysis and Related* In Silico* Computations for Designing Specific Target Binding Aptamers

An information-driven theoretical approach, EFBA, has been proposed for the computational design of aptamer templates based solely on structural information of the biomolecular targets [72]. This approach is free from some of the impediments of SELEX, such as the need to design randomized DNA/RNA libraries of appropriate complexity and aptamer length to provide candidates for binding with the specific targets. EFBA does not also require the labor- and time-intensive procedures to isolate aptamers from a large pool of candidates. The EFBA approach integrates the information-processing methods with the seed-and-grow strategy to determine the probability distribution of the nucleotide sequences that most likely interact with target structures. From this, we simultaneously determine sequences and the corresponding tertiary structures. The strategy adopted here consists of three steps: (1) firstly, determine the probability distribution of a preferred first nucleotide; (2) secondly, determine the probability distribution of preferred neighboring nucleotides given the probability distribution of the preferred nucleotide obtained in the previous step; and (3) thirdly, apply the entropic criterion to determine the preferred sequence length. When the relative entropy of the target-nucleotide fragment complex in the growth process is saturated, it indicates that the effect of the corresponding nucleotide on the interactions of the complex is at a global minimum. Therefore, the portion grown in the ME aptamer after the saturation step does not play an important role in the interactions and the preferred length *L* is the number of nucleotides grown before the saturation step. This way we determine the type of nucleotides and their minimum number in the sequence and thus the aptamer specific for a target gets created. As this is a review on general drug discovery and drug delivery aspect we shall avoid providing the detailed theories here but the readers are encouraged to read our journal cover taking paper [11], another recent paper by us [38], and other earlier reports in [60–68].

**Figure 4:**
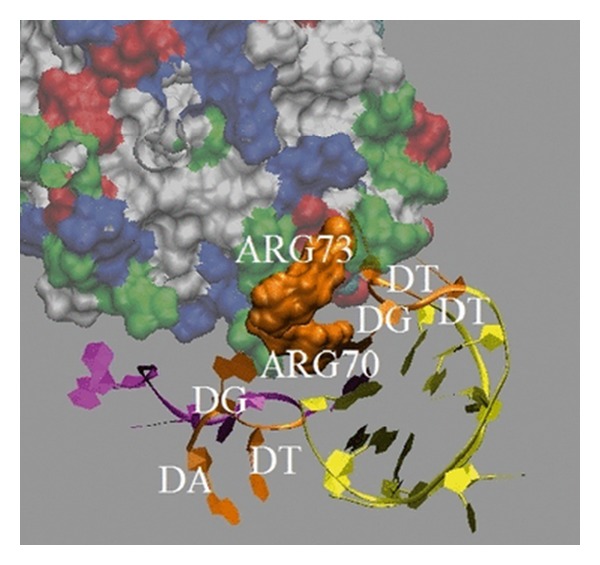
Binding modes of ME and SELEX thrombin aptamer are presented here (taken with due permission from [11]). The tertiary structure of the ME aptamer that binds to thrombin protein is shown in purple, and its binding location at 1 ns of the simulation was generated using visual molecular dynamics [8]. The structure of the SELEX aptamer colored by yellow and binding location at 1 ns of the simulation are shown for comparison. Both form nonbonded interactions with Arg70 and Arg73. The TGA and TGT loop from the ME and SELEX aptamers and residues Arg70 and Arg73 are shown in orange. The properties of residues in thrombin are color labeled. The acid residues are shown in red. The polar and nonpolar residues are shown in green and white, respectively. The remaining residues are represented in blue.

The* in silico* experiments (computations) are used to assess the binding affinity and selectivity of the predicted sequences. Two quantities, hydrogen bond interactions and binding free energy, are considered in our analysis. Computational/simulation details on how we analyze the hydrogen bond interactions and binding free energy estimation are provided in [11, 38]. To visualize computation results we just provide here the projection to show the binding modes of ME and SELEX thrombin aptamer in Figure 4 [11].

The computationally designed templates can further be modified theoretically and experimentally to optimize their binding affinity and additional properties related to biological suitability. The foundation of the EFBA method hinges on the integration of information processing with the seed-and-grow strategy. We have theoretically and experimentally investigated the applicability of the proposed approach using two specific targets, namely, protein thrombin and phospholipid PS [11]. Both of these initial studies support EFBA and indicate a promising advancement in theoretical aptamer design. Particularly, in the case of PS, two kinds of information, the total energy and interaction energy of PS and nucleotide fragments, were used to design aptamers. We then performed two sets of* in silico* studies using 10 ns explicit water molecular dynamics (MD) simulations using the PS and phosphatidylcholine (PC) head group as targets to investigate the binding affinity and specificity of the designed aptamers.

*Methods Utilized In Silico Studies of Aptamer-Lipid Binding.* The details have been presented in recent publications [11, 38]. A deeper theoretical understanding on the selectivity and binding affinity of aptamers was reported to be found from molecular dynamic (MD) simulations where Amber 11 with consideration of force field ff03 was used [73]. Following the Monte Carlo method, as initial structures five different relative locations and orientations have been randomly generated in every aptamer-phospholipid complex. Each location and orientation specific complex was energy minimized through the use of the steepest descent method for first ten cycles followed by a conjugate gradient for further 1000 cycles. During the period of equilibration of complex using explicit water TIP3P model, Langevin dynamics was then first applied during the process of heating up the system for 200 ps with targets being restrained with the use of a harmonic potential with a force constant *k* = 100 N/m to let the water molecules to be homogeneously distributed in the system. The pressure regulation was then introduced in the simulation to equilibrate solvent density for further 200 ps in addition to temperature regulation. After this a 10 ns explicit water MD simulation was performed at temperature 300 K and solution pH 7 for each complex. The targets were gently restrained with a harmonic potential having a force constant *k* = 10 N/m that was applied to the phosphorus in PS or PC. To further elucidate mainly the binding selectivity of the best possible candidate suggested from binding assays, 20 ns explicit water MD simulations were conducted [38]. Three quantities, the separation distance of centers of mass of aptamers and phospholipid, *d*_apt-lipid_, electrostatic (ES), and van der Waals (vdW) energy and solvent accessible area (SA), were utilized to analyze simulations [38].

**Figure 5:**
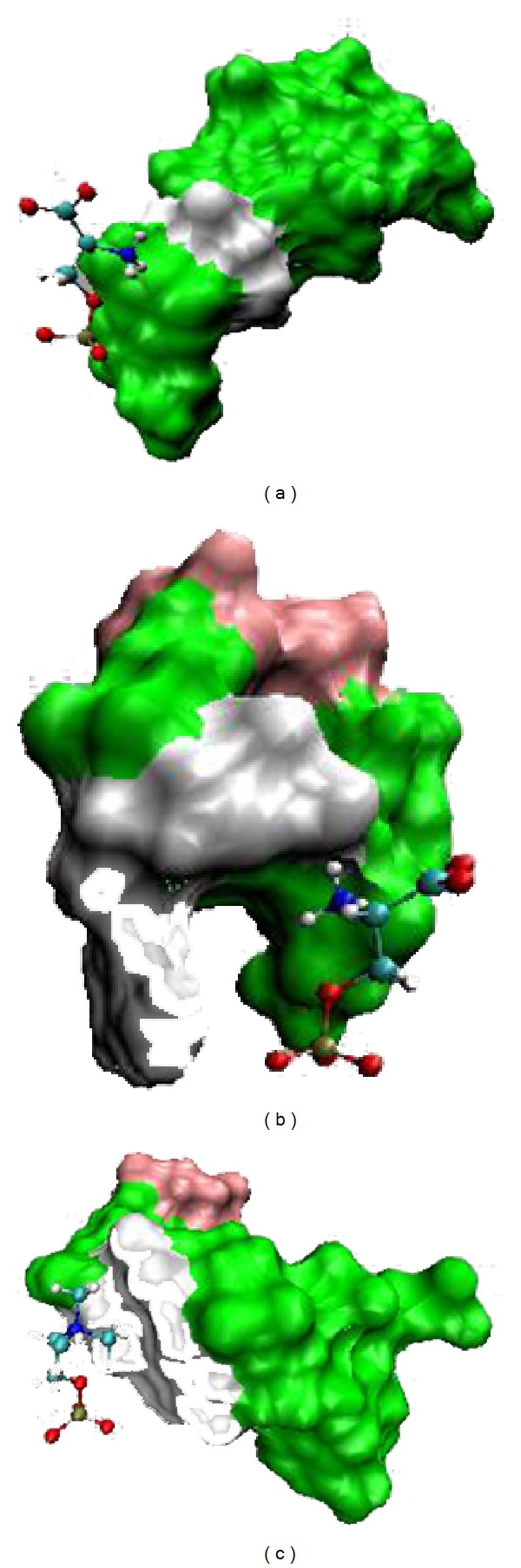
Surface representations of two ME aptamer structures at 5 ns are presented; A is shown in green, C in pink, and G in green. Panels (a) and (b) show the binding modes between two ME aptamers (PS-aptamer I and PS-aptamer II, resp.) and PS. Panel (c) shows the binding mode between PS-aptamer II and PC. The figure was rendered in VMD [43]. This figure is taken with due permission from [11].

At this preliminary stage, affinity was quantified by the mean distance of the center of mass between the target and aptamers. The binding modes of two top selected aptamers, 5′-AAAAGA-3′ (PS-aptamer I) and AAAGAC (PS-aptamer II), with PS and PC at 10 ns in the simulation are shown in Figure 5 (taken from [11]).

#### 2.4.2. *In Vitro* Experimental Validation

Designing aptamers theoretically is first step. MD simulation then validates computationally on the efficacy of the aptamers' binding to targets using energetics manipulations. But in reality experimental validation is seriously needed. In two subsequent publications [11, 38] our group has been successful in proposing the fluorescence based* in vitro* experimental technique to address the experimental aptamer-target binding properties.

**Figure 6:**
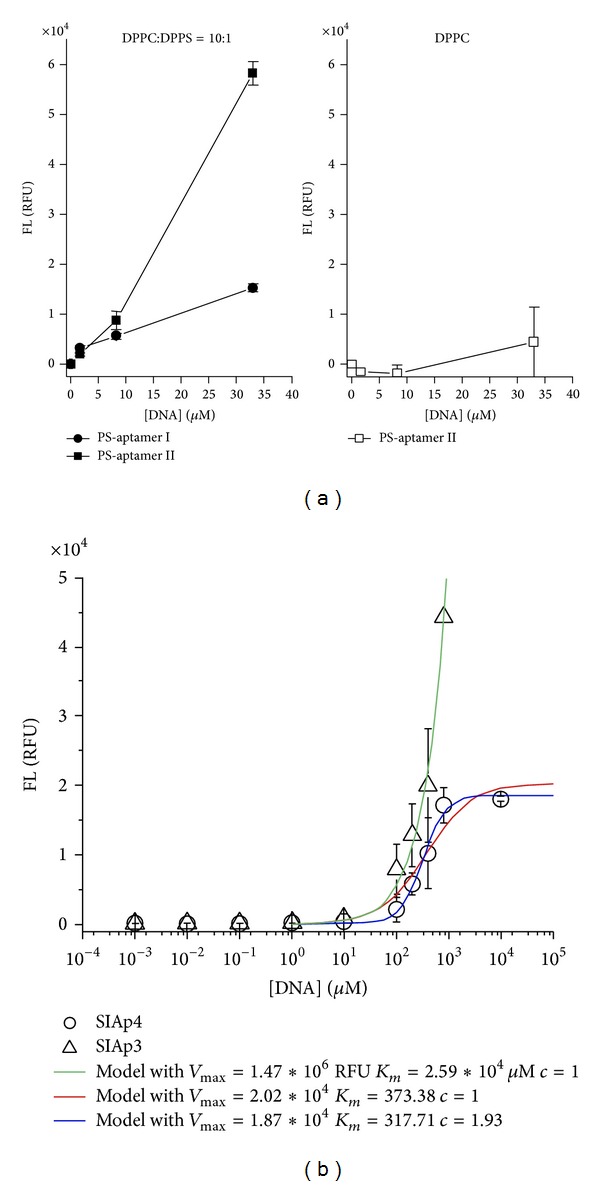
(a) Fluorescence (FL) measured in relative fluorescence units (RFU) versus aptamer concentration. Left panel: the selective binding of two designed DNA aptamers with liposomes containing PS. Right panel: the low, nonspecific binding of the designed DNA aptamer with liposomes containing only PC. The DNA concentration shown here is the actual concentration used in lipids/cholesterol in HEPES buffer. PS-aptamer I/SIAp1: AAAAGA, PS-aptamer II/SIAp4: AAAGAC [11, 38]. The data clearly show that PS-aptamer II/SIApIV binds to PS better than PS-aptamer I. This figure is taken with due permission from [11]. (b) The dissociation constant of SIAp4. Fluorescence versus aptamer (SIAp3: TAAAGA and SIAp4) concentrations. Only PS binding is measured. This figure is taken from [38].

The binding affinities between PS and two of the PS aptamers identified computationally were determined experimentally using methods [11] that are different from other popular methods [74] and are shown in Figure 6 [11]. Aptamers with fluorescent tags were synthesized by Integrated DNA Technologies, Coralville, USA. Phospholipid liposomes with a PC/cholesterol molar ratio of 2 : 1 were loaded into a 96-well plate in HEPES buffer. The test aptamer was then added from a stock solution in 1X Tris/EDTA (TE) buffer. The lipid/aptamer mixture was incubated in the wells for 40 minutes in dark condition. Supernatants (buffer and unbound aptamer) were then removed by washing three times with buffer, and 100 *μ*L HEPES buffer was added to each well. The fluorescence of the samples was measured with a FLUOstar OPTIMA plate reader (BMG Labtech GmbH, Offenburg, Germany). PC software version V1.30 R4 was used. The filters were set for excitation at 485 nm and for emission at 520 nm.

The designed aptamers were observed to bind to phospholipids in a lipid-specific manner. The aptamers showed a stronger binding affinity for PS liposomes than for PC liposomes (Figure 6(a)). The aptamer/PS binding is dependent on both DNA sequence and aptamer length and that aptamer II is found to be candidate specific for PS binding. These pilot studies provide an exciting proof of principal for the application of computational methods in aptamer design and provide the basis for the test of the use of our technique to discover target specific aptamers.

We then extended the binding assay studied on two sequences that are primarily detected as good PS binding agents to address their PS binding equilibrium conditions (see Figure 6(b)). As presented in [38], based on a single equilibrium reaction, one can derive the Hill equation, *FL* = *V*_max⁡_ *D* ^*c*^/(*K*_*m*_^*c*^ + *D* ^*c*^) with the Hill coefficient *c* = 1, and cooperativity of the complex; *V*_max⁡_ is the maximum fluorescence at equilibrium and *D* is concentration [75]. Furthermore, the parameter *K*_*m*_ represents the aptamer concentration required to have a half of the maximum fluorescence and it is an expression of affinity [75]. Therefore, the dissociation constant of aptamers can be estimated by *K*_*m*_. The models represented by red and green curves in Figure 6(b) are obtained by fixing the Hill coefficient to 1, resulting in a Michaelis-Menten type equation. The best model (blue curve) shows a dissociation constant around 317 *µ*M, which is slightly less than 373 *µ*M estimated in the red curve. Both are found to be far less than the value for SIAp3. Therefore, this study concludes that SIAp4 has the lowest value for the dissociation constant. SIAp4 has therefore been selected as the best candidate for PS binding. Our group is now dedicated to performing cell based assays using the sequence SIAp4 and its modified/engineered ones.

### 2.5. Chimeric SELEX and Chimeric Aptamers

A chimeric aptamer which is usually called a chimera is produced through the process in which natural recombination or chemical engineering is utilized to add two different aptamers together. Instead of two different aptamers sometimes an aptamer and other biomolecules are also added together to produce a chimera. Within a few years of the discovery of SELEX Burke and Willis [76] took pairs of aptamers that were previously selected to bind coenzyme A, chloramphenicol, or adenosine and fused them to create chimera. The chimera was found to retain some ability to bind both targets, but with reduced binding activity. Complex populations of recombined RNAs were also reported to give similar identical results. The investigators then applied dual selection pressure to recombined populations which yielded the combinations that were best suited to binding both targets. The chimeric aptamer selection is done by using chimeric SELEX in which the libraries are used for the production of chimeric aptamers with various features. Chimeric aptamers hold the potential activity of parent molecules and thus have the potential to show diverse combinatorial functions. The selected molecules taken from two parent molecular libraries are fused randomly to ensure that the chimera gets properties that are quite novel. Burke and Willis [76] got motivated from works of Joyce and colleagues who were the first to use point mutations through error-prone amplification and thus to optimize sequences for particular functions [77–81]. The duo thus came to conclusion that recombinatorial methods can also be applied to create new functions. The idea got raised also from the general consideration that nature uses a lot of genetic recombination to diversify the function of proteins. Chimeric SELEX that uses combination and recombination of functional RNAs may thus open the novel doors to generating bispecific molecules which may show greater varieties than the parent molecules' functions. Subsequent research and further developments have taken the chimeric aptamers into much higher stages [76, 82–100]. Various RNA, DNA, and antibody based chimeric aptamers have already been discovered with known mechanisms of actions on target biological structures. These structures, mostly in disordered states, are responsible for various diseases like cancer, HIV, and so forth, [76, 82–100]. As this review is about general aspects of aptamers I wish not to provide any details about this specialized field. However, I encourage readers with special interests in chimeric aptamers to study the above-mentioned references [76, 82–100].

## 3. Aptamers as Drugs

Most of the drugs target cell's various compartments where cell based diseases originate. The most critical role in cell function is played by cell signaling which controls the activation of cell's normal growth, raising cellular disorders, spreading diseases, and so forth. The cellular compartments and underlying mechanisms are therefore natural targets for drugs. Aptamers are invading fast in this area. I shall explain a few cases as examples here.

### 3.1. Macugen in Ophthalmology

Macugen has just got approved in ophthalmology as antiangiogenesis drug. This is first ever aptamer based drug. This is tried in the treatment of age-related macular degeneration [101, 102]. Macugen is also the first aptamer treatment approved by the FDA. Macugen inhibits the binding of 125I-VEGF to VEGF receptors Flt-1 and KDR expressed on porcine aortic endothelial cells (source: SomaLogic Inc.).

Macugen, a compound which was earlier (in 1998) taken by NeXstar with a name NX 1838, finally was pushed for early clinical development. In the SELEX based NeXtar discovery the first step was to place a pool of individual nucleotides in a test tube with the appropriate RNA or DNA polymerase to make aptamers that were poured over a column of VEGF to determine which ones would bind with fairly high affinity. Using the traditional SELEX technique those binders were then amplified, and more were made to find the ones with the highest affinity. Several cycles were repeated until the best binders were discovered. Thus finally the Macugen aptamer was discovered. Eyetech then tested the aptamer in its models to ensure that it behaved in the expected way, that is, to stunt vessel growth. It also performed standard toxicology and pharmacology tests. This is how Macugen has appeared as first successful aptamer based drug.

### 3.2. AS1411 as Anticancer Aptamer

Aptamer AS1411 is found to be the most advanced aptamer in the cancer setting, which is being administered systemically in clinical trials [103]. AS1411 is a 26-mer unmodified guanosine-rich oligonucleotide, which induces growth inhibition* in vitro*, and has shown activity against human tumor xenografts* in vivo*.

Various groups have studied the effects of AS1411 on cancer cell lines* in vitro* tests.

GROs (early names of AS1411), including AS1411 (formerly known as AGRO100), have shown growth-inhibitory properties, within less than a week, against a wide range of cancer cell lines,* in vitro*. GRO29A at a concentration of 15 *μ*mol/L induced growth inhibition of prostate (DU145), breast (MDA-MB-231 and MCF-7), and HeLa cancer cell lines [104]. A 10 *μ*mole/L concentration of either GRO29A or the unmodified GRO29AOH caused growth inhibition of the same panel of cell lines [105]. AS1411 was studied to evaluate the biologically active concentration* in vitro* [103]. The agent was incubated with a panel of human carcinoma cell lines, which included those derived from human prostate (DU145) and lung (A549) tumors. The IC50 of AS1411, the concentration required to elicit cell death in 50% of tumor cells, was reported at around 2 *μ*mol/L concentration range [103]. This same group also reported that a 7-day exposure of AS1411 at 10 *μ*mole/L dose had been found to induce more than 60% killing of three leukemia and two of three lymphoma cell lines.

In an* in vivo* biodistribution and antitumor activity study on mice models AS1411 is observed to be accumulated in tumor tissues [103]. This was followed by AS1411's preclinical toxicology studies in rats and dogs. In neither species was any significant toxicity observed, as determined by established clinical methods [103]. AS1411 was then selected for phase I clinical trial for 17 patients with advanced solid tumors (including 3 renal and 2 pancreatic) treated at the Brown Cancer Center (Louisville, KY) [103]. No serious toxicity effects were reported but promising activity findings was reported; one patient with renal cancer had a sustained partial response (at 16 months following treatment) and there were multiple cases of stable disease at 2 months (41% of patients, including three cases sustained for 6 months or more [103]).

AS1411 emerged as first nucleic acid based aptamer ever to be tested on humans for the treatment of cancer. The mechanism underlying its antiproliferative effects in cancer cells seems to involve initial binding to cell surface nucleolin and internalization, leading to an inhibition of DNA replication.

A few years earlier it was reported in a cell based study that plasma membrane nucleolin is a functional receptor for AS1411 in MV4-11 leukemia cells [106]. In an earlier study the same group reported that the plasma membrane nucleolin targeting AS1411 had been found to destabilize B-cell lymphoma-2 (BCL-2) messenger RNA in human breast cancer cells [107]. This destabilization of BCL-2 mRNA may be one mechanism by which AS1411 induces tumor cell death. More cell based studies are needed to confirm this claim. But it can be said quite confidently that AS1411 or its modified versions perhaps have potential use in cancer treatment.

### 3.3. DNA Aptamers in Apoptosis Detection

The EFBA [11] technique has proposed a few aptamer sequences for PS binding. It is discussed above in Section 2.4 that the PS aptamers are especially discovered for the purpose of detection of PS externalization in apoptosis [44, 45, 108–111].

The induction of programmed cell death or apoptosis is both a desired outcome of cancer therapy and a potential target allowing imaging and modulation of therapeutic effects. Apoptosis is a vital, highly regulated, natural process that contributes to the development and maintenance of humans and animals [112, 113]. Apoptosis plays multiple roles in the normal development of organisms extending from embryonic development to the maintenance of normal cell homeostasis [114–116]. Malfunction of the apoptotic process leads to the proliferation of many types of cancer due to inadequate control of cell homeostasis. It is recognized that evasion of apoptosis is one of the hallmarks of cancer development and progression [117]. There are a number of related cellular processes observed during apoptosis including PS externalization, caspase activation, chromatin and nucleus condensation, reduction in cytoplasm volume, and DNA degradation [115]. Drugs targeting and modulating apoptosis have a recognized potential in cancer diagnosis and therapy.

There are two main apoptotic pathways: the death receptor (extrinsic) pathway and the mitochondrial (intrinsic) pathway [118]. The death receptor pathway (MAPK) is activated by the binding of FAS or TRAIL ligands to their receptors (DR4/5), stimulating receptor aggregation. In the mitochondrial pathway, prosurvival signaling through AKT activation stimulates phosphorylation of BAD. This allows B-cell lymphoma (BCL 2) protein (encoded by the BCL 2 gene) to exert its antiapoptotic effects by blocking proapoptotic proteins BAX, NOXA, and so forth. However, dephosphorylated BAD blocks BCL 2 by heterodimerization (BAX/BCL 2), which allows proapoptotic proteins to form pores in the mitochondria. This process then releases apoptogenic factors from the mitochondrial intermembrane space, including cytochrome C (Cyt C), APAF1, and caspase 9. These factors form the so-called apoptosome, which stimulates apoptosis through caspase 3 cleavage. Conventional studies have shown that the BCL 2 protein family is one group of gene products that govern the initial phase of apoptosis [119]. Both antiapoptotic (BCL 2 protein) and proapoptotic (BAX) family members, whose solution structures have been experimentally determined [120–122], are potential drug targets in cancer treatments [123]. The multiple sequence alignment for six BCL 2 family proteins presents common motifs among these proteins [122]. Two potent small-molecule inhibitors (ABT-737 and ANT-263) designed to inhibit BCL 2/BCL-xL proteins have been described recently [123–125]. This inhibition is likely to help overcome the BCL 2 protein-induced antiapoptosis activity [124]. A strategic study can be suggested for apoptosis modulation in cancer treatment by considering an appropriate combination of “regulators.” The regulators would act at different proteins which are responsible for triggering and/or inhibiting apoptosis. Here specifically we can consider a set of regulators consisting of inhibitors for BCL 2 protein (antiapoptosis) and enhancers for BAX's proapoptosis effect. The modulation of cancer treatment however can be monitored looking at the lipid functions in membranes.

**Figure 7:**
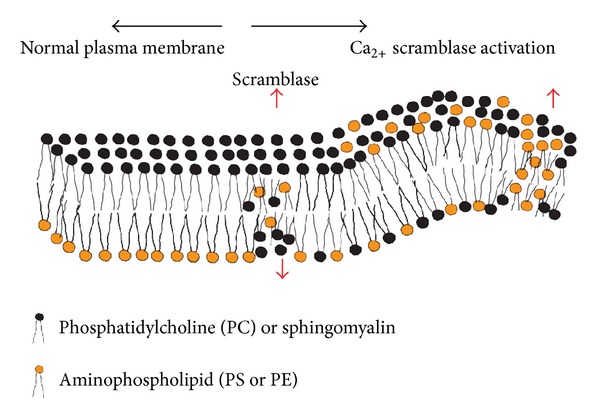
PS externalization [44, 45] in the apoptotic cell membrane. Early in the apoptotic process there is a rapid redistribution and exposure of phosphatidylserine (PS) on the cell surface mediated by the enzyme scramblase. Due to perhaps specific lipid properties, for example, especially intrinsic curvature, the PS concentration varies between inner and outer leaflets on lipid monolayers in a membrane. PS is normally restricted to the inner leaflet of the lipid bilayer by an ATPdependent enzyme called flippase (translocase). Flippase, in concert with a second ATP-dependent enzyme, floppase, that pumps cationic phospholipids such as phosphatidylcholine (PC) and sphingomyelin to the cell surface, maintains an asymmetric distribution of different phospholipids between the inner and outer leaflets of the plasma membrane. This figure is redrawn [46] in light of the model diagram and description of the general apoptosis process presented in [44, 45].

For diagnostic purposes, the redistribution of PS between inner and outer plasma membranes can be targeted (see Figure 7 [46]) as recently described in detail [108], (PS externalization), which has been shown as an early marker of apoptosis. PS externalization is specific to apoptotic cells with the exceptions of activated platelets and erythrocytes. PS externalization therefore is an attractive target to detect apoptosis [44, 45, 109] and to provide an early indication of the success or failure of therapy for cancer patients in a clinical setting. Lahorte et al. provide a thorough overview in apoptosis-detecting radiotracers [110]. Currently, annexin V is considered to be the most promising agent in clinical applications [44]. Several groups have prepared  ^18^F labeled annexin V by different approaches to be used with positron emission tomography (PET) because of its higher resolution and more quantitative imaging [45, 126–128]. However, the value of these radiopharmaceuticals for human use remains to be determined. Detection of PS externalization can be a very good alternative method for apoptosis detection purposes. We are working on developing a vital method to find correct PS aptamers using nucleic acid oligos [11] that are less toxic, easy to synthesize, and cheap alternative to hazardous other candidates. We are also in the process of developing further to scroll down our search for best candidate aptamer that will target the PS externalization in apoptotic cells. The readers are encouraged to read our recent publication [38] and keep their eyes open to catch our subsequent papers as they become available. From the discussion presented here it is to be said that a set of aptamers that would regulate apoptosis by acting upon BCL 2 proteins and detect the apoptosis by acting upon PS externalization would serve as a set of theranostic (therapeutic + diagnostic) drugs.

### 3.4. Aptamers in Alzheimer's Disease

Despite some promising scopes for aptamers to be used in many diseases the application of aptamers in age related Alzheimer's disease (AD) [129] is not so far confirmed. Aptamers' use has remained scarce in amyloid research, including AD. AD is a progressive neurodegenerative disease believed to be caused by neurotoxic amyloid *β*-protein (A*β*) oligomers. A*β* oligomers are therefore attractive targets for development of diagnostic and therapeutic reagents. Rahimi et al. [130] used covalently-stabilized oligomers of the 40-residue form of A*β* (A*β*40) for aptamer selection. Despite gradually increasing the stringency of selection conditions, the selected aptamers did not recognize A*β*40 oligomers but reacted with fibrils of A*β*40, A*β*42, and several other amyloidogenic proteins. Aptamer binding to amyloid fibrils was found to be RNA-sequence-independent. The study suggested that aptamers for oligomeric forms of amyloidogenic proteins could not be selected due to high, nonspecific affinity of oligonucleotides for amyloid fibrils but that they could serve as superior amyloid recognition tools. In a subsequent study Rahimi and Bitan [131] attempted to select aptamers using SELEX for covalently stabilized oligomeric A*β*4021 generated using photo induced cross-linking of unmodified proteins (PICUP) [132, 133]. Similar to other findings [134–136], these aptamers reacted with fibrils of A*β* and several other amyloidogenic proteins likely recognizing a potentially common amyloid structural aptatope [130]. A lot of more basic studies are still needed on aptamer's use in Alzheimer's disease treatment.

### 3.5. Cases of Other Diseases

SELEX has been extensively used to search for aptamers that would target several structures. Theoretical and computational techniques including EFBA are also in use to search for right aptamers for binding with right structures that are responsible for diseases. We have extensively described the generation of aptamer-based probes for detecting and for regulating apoptosis with potential relevance in cancer treatment. In this context the early detection of response to therapy and the possibility to augment treatment by the use of aptamers would have huge clinical benefits. We see this as the primary health-related potential. A parallel diagnostic and therapeutic approach provides both confidence in treatment and understanding the progress. However there are many other potential benefits to be derived from a molecular imaging probe for apoptosis in other human health situations (e.g., transplantation) and human pathologies including neurological disorders and cardiovascular disease. In addition, we can envision a role for an animal-based apoptosis imaging model in drug development of novel aptamer-based therapy. Additionally the computational design of aptamers would not be restricted to targets relevant to apoptosis but could be employed in any situation where a well characterized target is known. EFBA [11] may appear as a universal technique to design aptamers for any target with some basic known information.

#### 3.5.1. Pharmacokinetics of Aptamers

**Figure 8:**
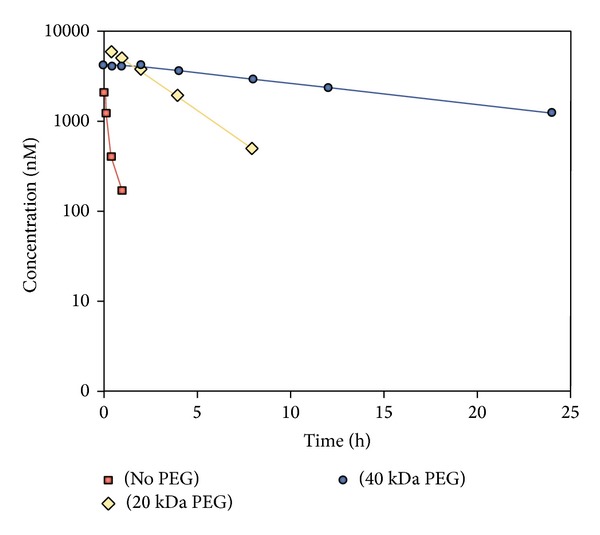
The pharmacokinetics of aptamers conjugated to different molecular mass pegs (taken from [47]).

In this Section 3, we have addressed so far a few example cases where aptamers are claimed to be possible drug candidates. Aptamers are found to be utilizable in both therapeutic and diagnostic applications. The success however relies on many factors that need to be checked* in vitro* and* in vivo* tests. Computational pharmacokinetic tests are also powerful and generally used to address drug's target specific binding [11, 38]. An appropriate* in vitro* multitarget drug distribution assay (under construction by my group) may help the* in silico* assay to be verified and thus provide enough information for the* in vivo* pharmacokinetics of drugs. A recent review by Ellington group [47] has addressed some aspects of this quite specifically. The half-lives of unmodified nucleotide aptamers in the blood are of the order of 2 minutes [137]. Thus short aptamer stability is a serious issue. To overcome this problem chemical modifications are proposed to be incorporated into the nucleotide sugars or internucleotide phosphodiester linkages. This increases serum half-life. Endogenous serum nucleases are reported to have higher degradation rates when cleaving at pyrimidine residues [138]. Now SELEX consisting of completely modified oligonucleotide compositions is a reality [139]. Due to small size aptamers' renal filtration is a big issue. Most of the aptamers are within 50 nucleotides length that fall within renal filtration range. Through polymer conjugation, for example, 40 kDa PEG-conjugated aptamers largely reduces the renal filtration (see Figure 8 [140]).

In Figure 8, the pharmacokinetic profiles of 39-mer 2′-deoxy purine, 2′-*O*-methyl pyrimidine composition aptamers have been presented. These aptamers were unconjugated or conjugated to either 20 kDa polyethylene glycol (PEG) or 40 kDa PEG and administered intravenously to CD-1 mice (*n* = 3 per time point) at 10 mg per kg [140]. A clear contrast is visible between three conditions as presented in Figure 8. An appropriate pharmacokinetic profile is therefore needed to be developed for obtaining controlled aptamer distribution among targets of interests.

## 4. Aptamers as Drug-Carriers

Most of the cell based disorders or diseases require drugs to be physically delivered into the disease sites. These sites exist mostly in various regions of intracellular or extracellular proteins, membrane proteins, lipids, and mitochondria. Cell targeted drugs are often released in the extracellular regions by plasma fluid. Nanoparticle delivery of drugs is also very popular. Drugs then need to be delivered beyond membrane barrier which is pretty tricky. General drug diffusion across membrane barrier due to positive drug concentration gradient between extracellular and intracellular regions is mostly used for drug delivery mechanism. A membrane-based engineering may result into superior delivery mechanism (see [46, 141]). But in this case the possible aptamer-lipid interactions [11] may appear as general background creating factors. Besides lipids there are other aptamer interacting agents that may appear in the cell that may be targeted in the case of aptamer-mediated drug delivery. It is now evident that the perfect target for aptamer-mediated delivery is one that is highly expressed on all target cells, is efficiently internalized, and is not expressed on the surface of nontarget cells [36]. I shall discuss on a few of all these aptamer-mediated drug delivery mechanisms here.

### 4.1. Aptamers and Nanoparticles

Aptamers due to its specific lipid binding properties [11] may also be considered as nanoparticles besides being used as lipid bound drugs. We (Tseng et al. [11] and Ashrafuzzaman et al. [38]) have recently shown that DNA aptamers may bind to specific lipids, for example, PS on the outer membrane layer in the apoptotic PS externalization region. This means these aptamers avoid binding to PC which accounts for most (60%>) of membrane constructing lipids. For details see chapter 4, Membrane Biophysics [46]. The mentioned studies also discover a set of longer DNA aptamers that usually bind to neither PS nor PC. The poor lipid interacting aptamers [11, 38] may be modified for use as nanoparticles that can diffuse across cell membrane by repelling lipids in a way identical to that of silica nanoparticles, explained in [46, 142]. The mechanism explained here requires direct aptamer- (instead of silica-) based test on a model membrane system. We are performing the investigations using theoretical, computational, and experimental techniques. A manuscript with promising results is under preparation.

### 4.2. Transport across Membrane: Natural Diffusion

#### 4.2.1. Analytical Expressions and General Understanding

In a recently published book “Membrane Biophysics” [46] we have developed a theoretical analysis on how membrane's selective transport phenomena experience energetic regulation. This regulation is due to both physical membrane properties and the physical coupling energetics between membrane constituents and the agents to be transported. A cell membrane's primary role is to serve as a barrier against the solutes trying to diffuse across it. With the physical presence of membrane the cytoplasm maintains a different composition from the materials surrounding the cell. The membrane is very impermeable to ions and charged molecules. It is permeable to small molecules in the cell environment in inverse proportion to their size but in direct proportion to their lipid solubility. The membrane also contains various pumps and ion channels made of specific membrane proteins that allow concentration gradients to be maintained between the inside and outside of the cell. For example, the cation pump actively extrudes sodium ions (Na^+^) from the cytoplasm and builds up a concentration of potassium ions (K^+^) within it. The major anions inside the cell are chlorine ions (Cl^−^) and negatively charged protein molecules, the latter of which cannot penetrate the membrane. The presence of the charged protein molecules leads to a buildup of electroosmotic potential across the membrane and thus shows some important regulatory role. Action potentials resulting from the transient opening of Na^+^ or calcium ion (Ca^2+^) channels depolarize the membrane, followed by an opening of K^+^ channels leading to repolarization. All these chemical and physical properties control the natural transport through membranes. Ions like Na^+^, K^+^, Cl^−^, Ca^2+^, and so forth find some cellular processes to cross through the membrane. But external agents like nanoparticles with unique physical properties find no direct way to cross through the plasma membrane. The membrane thickness is about 3 nm for hydrocarbon-free bilayers [143, 144] and 4-5 nm for hydrocarbon-containing bilayers [145]. Lipid cross sectional area in a monolayer of a membrane is of the order of 0.6 nm^2^ [146]. Nanoparticles are supposed to be of the order of a few nm in dimensions especially those aptamers claimed to have some kind of nanoparticle criteria (discussed in Section 4.1). Membrane is liquid crystalline structure where the lipids experience continuous lateral movement in the plane of the membrane monolayer. But the lipid-lipid separation is always maintained at about 0.8 nm [146] unless any membrane disorder occurs due to the effects of any membrane residing agents. These agents may be membrane proteins or external ones like drugs, antimicrobial peptides, and so forth. Despite a possibility of very slow diffusion of the nanoparticles into the cellular interior due to the possible high concentration of nanoparticles on the membrane surface a satisfactory level of nanoparticle transport cannot always be ensured. For this we need to discover a controlled transport mechanism or a unique “nanotechnology” (to be discussed later in Section 4.3) which must involve the consideration of the physical and chemical properties of both membranes which needs to be crossed through and the nanoparticles to be delivered. Due to the cell membrane's natural barrier against most of the agents except a very few ions residing in the cellular environments we need to discover the mentioned novel nanotechnology involving a few membrane active agents. These agents may instantaneously destroy the membrane's barrier properties in a controlled manner and allow the nanoparticles to cross through the membrane. By compromising the membrane's barrier properties the membrane's transport properties may be modulated. But in case of natural nanoparticle transport across membrane the transmembrane flow depends mainly on two external agents. Firstly, the difference of hydrostatic pressure between the two fluid compartments on either side of the membrane. This pressure gradient is physiological and it exerts natural effects on every particle flow across the membrane. The second agent is the gradient of solute concentration between the two compartments separated by membrane. Although the physiological pressure gradient is organ specific and the transport of particles naturally needs to deal with this but the second agent plays like an input condition in the case of nanoparticle transport across the membrane. With an appropriate technique we can control the number density of nanoparticles just outside the membrane (extracellular regions) and consider that in the beginning the number density of nanoparticles beyond the membrane (intracellular regions) is zero. Besides these two agents, there is a very important mechanism needed to be considered. This mechanism determines the free energy of nanoparticles while crossing the membrane. This discussion is dedicated to mainly understanding the physical phenomena of membrane transport of nanoparticles, that is, the energetics of nanoparticles inside the membrane. Based on this understanding we wish to develop a novel nanotechnology to deliver nanoparticles beyond membranes.

Understanding of the membrane transport of nanoparticles requires specific information about the geometry and constituents of the membrane which determine the membrane partition against or in favor of transport of any particles across the membrane [46]. Membrane partition coefficient (*К*_*m*_) and the particles' experimentally measurable free energy of interaction with membrane (Δ*G*_*m*_) are related through the following equation:

(1) $$\begin{array}{c} \Delta G_{m}=-RT\ln\left({К}_{m}\right). \end{array}$$

Here *R* is universal constant and *T* is the absolute temperature.

It is very important to mention that the values of *К*_*m*_ play important role in determining the membrane permeability (*P*_*m*_) of any drug transport across membranes. A linear relation is generally accepted between *P*_*m*_ and *К*_*m*_ [147] which follows:

(2) $$\begin{array}{c} P_{m}=\frac{D_{m}{К}_{m}}{L}, \end{array}$$

where *D*_*m*_ is membrane diffusion coefficient of the particles. *L* is the bilayer membrane thickness. The membrane permeability coefficient of particles (*P*) or drugs which is the linear velocity (nm/s) of the drugs through the membrane is in fact the rate of particle transport through the membrane. Derivation of *P* requires very accurate consideration of all components of *D*_*m*_ and *K*_*m*_. We can instead consider the fractional release of the particles by membrane into the cellular interior considering that the number density of particles (*ρ*_np,ext_) at the entry level into the membrane (just outside of the membrane) is known. Let us assume that the number density of particles at the release level beyond the membrane (just inside cell) is *ρ*_np,int⁡_; we then can propose an analytical relation between these two number densities following the equation

(3) $$\begin{array}{c} \rho_{\text{np},\mathrm{int}}=f\left(L,H,\varepsilon_{m},\rho_{\text{np},\text{ext}}\right). \end{array}$$

By *f*(*L*, *H*, *ε*_*m*_, *ρ*_np,ext_) I have meant that this is a function of *L*, *H*, *ε*_*m*_, and *ρ*_np,ext_. Here *H* is the average Hamiltonian of any nanoparticle inside the membrane and *ε*_*m*_ is the relative membrane dielectric constant. The most important component *H* can be expressed as follows:

(4) $$\begin{array}{c} H=U_{\text{np-lip}}+T_{\text{np}}. \end{array}$$

Here *U*_np-lip_ stands for the sum of all kinds of interactions felt by a nanoparticles and *T*_np_ stands for the nanoparticle's kinetic energy while being inside membrane. If we use no other membrane proteins but the lipids and membrane stabilizing hydrocarbons to form a membrane (e.g., see [148, 149]) we can consider the expression of *U*_np-lip_ to be following this relation:

(5) $$\begin{array}{c} U_{\text{np-lip}}=U_{\text{ES}}+U_{\text{vdW}}+U_{\text{mechanical}}+U_{\text{hydration}}. \end{array}$$

*U*_ES_ is the resultant of the electrostatic interaction (ES) energies and *U*_vdW_ is the sum of van der Waal's (vdW) interaction energies of the nanoparticle with lipids in the pathways of the nanoparticle. *U*_mechanical_ is the energy arising from the mechanical properties, namely, the membrane elasticity and membrane monolayer curvature. *U*_hydration_ is the contribution due to the hydration energy. I have ignored the interaction energies due to the interactions with hydrocarbons and any other possible sources for the sake of simplicity of the calculation.

A few chapters in the book “Membrane Biophysics” [46] have been dedicated to addressing how the mentioned energy components can be derived if the structural and charge properties of the participating components are known. Using this theoretical analysis one can gain some preassumption whether certain aptamer based drugs or drug carriers can at all get transported across the cell membrane to reach out to the cell's interior regions.

Certain nanoparticles may disrupt membrane's barrier properties instantaneously which raises possibility for various agents to reach in the cellular interior regions. Nanoparticles that are designed to deliver drugs beyond membrane may interact with membrane itself and create stable or temporary holes or defects there (see chapter 4, Membrane Biophysics [46]). The complete loss of a region of the plasma membrane where the lipids are removed can be referred to as the word hole or pore. The mentioned complete loss of lipids is usually a transient phenomenon. Due to the liquid crystalline [150] nature of the lipid membrane and the presence of strong statistical mechanical effects on membrane dynamics the nearby lipids fill in the gap quickly. The membrane disruption depends on size and structure of nanoparticles [151]. For a few reference studies addressing possible nanoparticle disruption of the membrane readers are encouraged to read chapter 6, Membrane Biophysics [46].

#### 4.2.2. A Few Practical Cases as Examples

Recently, a study on aptamer-based tumor-targeted drug delivery for photodynamic therapy has been performed by Shieh et al. [152]. Here a specialized G-rich DNA structure, G-quadruplex, was studied for its special physical characteristics and biological effects. This reference has reported a novel strategy of using G-quadruplex as a drug carrier to target cancer cells for photodynamic therapy (PDT). A G-quadruplex forming AS1411 aptamer was reported to have properties which could help to be physically conjugated with six molecules of porphyrin derivative, 5,10,15,20-tetrakis(1-methylpyridinium-4-yl)porphyrin (TMPyP4), to fabricate the aptamer-TMP complex. The TMPyP4 molecules in the complex had been identified to bind tightly to the aptamer by intercalation and outside binding. The effect of the G-quadruplex structure as a carrier for the delivery of TMPyP4 into cancer cells by nucleolin-mediated internalization was investigated. The results showed that the aptamer-TMP complex exhibited a higher TMPyP4 accumulation in MCF7 breast cancer cells than in M10 normal epithelium cells. After being treated with light for 180 s, the photodamage in MCF7 cells was larger than in M10 cells. These results indicated that the TMPyP4 delivery and uptake were mediated by the specific interaction of the aptamer-TMP complex with nucleolin on the cellular surface and that the use of the AS1411 aptamer as a drug carrier may be a potential tactic in cancer therapy. Thus the aptamer AS1411 which has been previously addressed as an anticancer agent is found to have capacity to also be used as a drug carrier for specific purposes.

**Figure 9:**
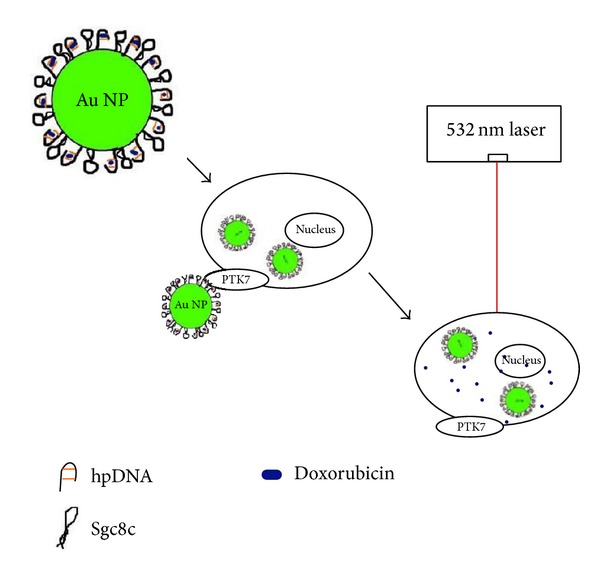
Model diagram on an aptamer/hairpin DNA-gold nanoparticle (apt/hp-Au NP) conjugate for targeted delivery of drugs. This model diagram has been redrawn in light of the figure presented in [48].

In another study by Luo et al. [48] a smart drug carrier, an aptamer/hairpin DNA-gold nanoparticle (apt/hp-Au NP) conjugate for targeted delivery of drugs, had been devised. The DNA aptamer sgc8c, which possesses strong affinity for protein tyrosine kinase 7 (PTK7), abundantly expressed on the surface of CCRF-CEM (T-cell acute lymphoblastic leukemia) cells, was assembled onto the surface of Au NPs. The repeated d(CGATCG) sequence within the hpDNA on the Au NP surface was used for the loading of the anticancer drug doxorubicin. After optimization, doxorubicin molecules were successfully loaded onto the AuNP (13 nm) surface. The binding capability of aptamer/hp-Au NP conjugates toward targeted cells was investigated by flow cytometry and atomic absorption spectroscopy, which showed that the aptamer-functionalized nanoconjugates were selective for targeting of cancer cells. A cell toxicity (3-(4,5-dimethylthiazol-2-yl)-5-(3-carboxymethoxyphenyl)-2-(4-sulfophenyl)-2H-tetrazolium, MTT) assay also demonstrated that these drug-loaded nanoconjugates could kill targeted cancer cells more effectively than nontargeted (control) cells. Most importantly, when illuminated with plasmon-resonant light (532 nm), doxorubicin: nanoconjugates displayed enhanced antitumor efficacy with few side effects. The marked release of doxorubicin from these nanoconjugates in living cells was monitored by increasing fluorescence signals upon light exposure.* In vitro* studies confirmed that aptamer-functionalized hp-Au NPs could be used as carriers for targeted delivery of drugs with remote control capability by laser irradiation with high spatial/temporal resolution. A model diagram explaining the mechanisms has been presented in Figure 9.

Recently, a review on tumor-targeted drug delivery with aptamers has demonstrated the aptamer based drug delivery aspects quite in detail [153]. Here aptamer-based delivery of chemotherapy drugs such as doxorubicin, docetaxel, daunorubicin, and cisplatin, toxins like gelonin and various photodynamic therapy agents, and a variety of small interfering RNAs have been discussed in detail. The report summarizes that although the results are promising much to be done before aptamer-based drug delivery can reach clinical trials and eventually the day-to-day management of cancer patients.

### 4.3. Transport across Membrane: Through Transiently Constructed Ion Pores

**Figure 10:**
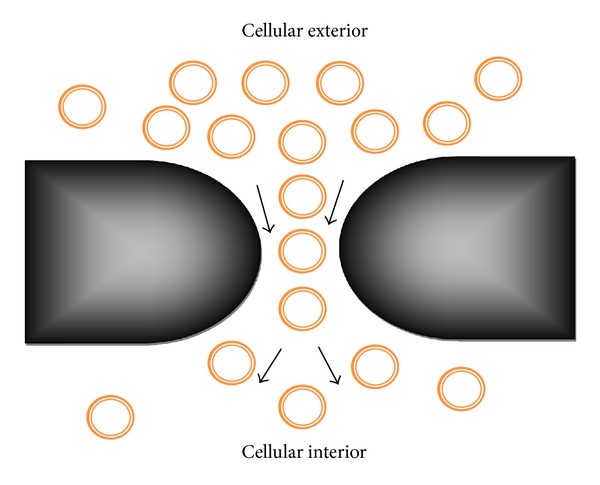
Nanoparticles with nonlipid interacting criteria, for example, long DNA aptamers [11], naturally diffuse through the type of toroidal pores where the opening region inside the pore consists of no pore inducing agents. The absence of pore inducing agents in the opening regions helps nanoparticles experience no considerable direct interactions with the agents. We have possibly observed such a type of pore induced by chemotherapy drugs. Beside chemotherapy drugs, a possible search for a better set of agents, for example, any natural or synthetic antimicrobial peptides or biomolecules which might create the type of pore as mentioned here, should be a meaningful investment. This will ensure a combination therapy at low cytotoxicity.

Chemotherapy drugs (CDs) are found to induce lipid-lined toroidal pores in lipid membranes. The model diagram is described in chapter 4 [46]. Many other antimicrobial peptides like magainin, melittin, colicin, and so forth also induce toroidal pores [154–156]. Some general pores despite being protein lined transport materials through them. Gramicidin A channels [157] are especially mentionable. The chemotherapy drug induced toroidal pores show some unique biophysical characteristics. The first ever observed triangular conductance events (explained details in chapters 4 and 5, Membrane Biophysics [46]) may appear with very important ingredients which would help them find use in developing a novel nanotechnology. The induction of independent triangular conductance events suggests that the conductance in a single event is not constant but increases/decreases spontaneously over the time interval comparable to the low millisecond (ms) order “lifetime” of any specific conductance event. A spontaneous transition or a time-dependent current fluctuation between random current levels in CD-induced conductance events actually suggests the existence of a pore whose cross-sectional area fluctuates with time. This has been schematized with having a lipid-lined toroidal pore (see Figure 10).

The chemotherapy drug induced broken regions of membrane or the lipid-lined toroidal pores behave exactly like regions without the hydrophobic membrane core. Any material which does not like membrane environment (specifically the membrane inner hydrophobic core) may find these pores to be favorable regions of interest for passage. It is predicted that the pore inducing agents reside behind the lipids. Any material passing through the pore can therefore avoid any kind of direct interactions with the pore inducing agents. It is mentionable that certain nonlipid interacting nanoparticles like silica [142], long DNA aptamers [11, 38], for example, are likely to experience weak interactions and to show weak binding to lipids while others like polycationic polymer nanoparticles, for example, are likely to experience stronger interactions and to show better binding with lipids. Nanoparticles with neutral or nonbinding attitude towards lipids can easily be driven through the chemotherapy drug induced broken (toroidal pores) type regions. This is schematically diagrammed in Figure 9. With the absence of considerable binding with membrane the nanoparticles can easily diffuse to the cellular interior regions (for detailed theoretical analysis see above [46]). Induction of lipid-lined toroidal pores [13] may therefore be a mechanism for nanoparticle carrying delivery of drugs into cellular interior regions.

The proposed novel nanotechnology [46] heavily depends on consideration of charge and geometry of nanoparticles, and dimension and stability of the pores transporting these nanoparticles. As the proposed pore is “lipid-lined” type the lowest possible value of the cross section of a pore is as small as 0. From there the cross section can increase to any value. The pore cross section of lipid-lined toroidal pore changes through increase or decrease or following a back-and-forth change of the value of the area of cross section of the pore [13, 158]. This ensures the possibility of free transport of nanoparticles with any dimension which can even be as low as the dimension of the order of a lipid head group. This will be able to bring the freedom of choosing nanoparticles of any size for a drug delivery into the cellular interior regions. Drug carrying smaller size nanoparticles will require induction of smaller pores and the particle diffusion may also be faster due to lower value of nanoparticle inertia. This will thus require low stability (~pore lifetime) for the pore. This novel nanotechnology should be able to deal with drug delivery targeting at the cellular interior regions. Certain aptamers may serve as such nanoparticles. A real application exists in treatment of various types of cell based diseases like cancer, Alzheimer's, and any other disorders therein. Besides, imaging of the cellular interior regions can also be performed using our novel nanotechnology. Here cell exterior versus interior region distribution of aptamer based nanoparticles with fluorescence tags attached can be tracked down. Our nanotechnology can therefore find its use in both cell based diagnosis and therapeutic applications which will enhance our understanding of various cellular processes that determine perhaps the definition of life.

## 5. Conclusion

I have outlined a detailed analysis on major techniques developed so far to design aptamers starting from the discovery of SELEX. Theoretical* in silico* and* in vitro* methods to design aptamers as well as computational* in vitro* and* in vivo* experimental validations have been used to understand the potencies of the developed techniques. All the aptamer design techniques have been under thorough scrutiny by many groups all over the world. Successes have been found in many cases. SELEX based discovery of Macugen as antiangiogenesis drug has now been approved in ophthalmology. Aptamers are now rigorously explored for use in various other diseases like cancer, Alzheimer's, and so forth. I have provided brief information on all these in this review. Drug delivery is another important area where aptamers have started finding their roles. Less toxicity and more target specific binding, these are the two basic criteria in designing aptamers for certain target structures. Our newly devloped EFBA based aptamer design technique may be utilized for designing aptamers specific for disorders whose structures are somewhat known. As we understand more of the biophysical states of diseases various aptamer design techniques will find their use in discovering more and more aptamer based drugs specific for the disease states. With the developed understanding of the drug pathways and biophysical aspects of off-target interactions new aptamers will also be designed for their use as drug-carriers. As the aptamer design techniques become trustworthy pharma companies [159, 160] may even adopt them and start offering commercial services on designing target specific aptamers for both academic researchers working on understanding aptamer based therapeutics and scientists working in companies for commercial development of aptamer based drugs. Overall, this review will help create a platform to understand the designing process of aptamers as drugs for specific diseases. These aptamers may also be used as diagnostic tools to understand the efficacy of the use of other drugs and as drug carriers for many drugs. Various techniques have also been explained here to address the aptamers' cytotoxicity aspects. Thus it will help drug discovery scientists working in academia and pharmaceutical companies as an important reference article.

## Figures and Tables

**Table 1:** Available aptamers currently under considerations.

Name of the aptamer	Primary target of the aptamer	Status
Macugen	VEGF	Approved [22]
AS1411	Nucleolin	Phase II [25, 26]
REG1	Factor Ixa	Phase II [29, 30]
EYE001	VEGFR	Phase II/III [47, 49]
LY2181308	Survivin mRNA	Phase III [50, 51]
E_2_F decoy oligonucleotides	Mesangial cells	Phase III [52, 53]
ARC1779	Vwf	Phase II [31]
NU172	Thrombin	Phase II [32]
E10030	PDGF	Phase II [23]
ARC1905	C5	Phase I [24]
NOX-E36	MCP-1	Phase I [27, 33]
NOX-A12	SDF-1	Phase I [27, 28]
NOX-H94	Hepcidin	Phase I [21]
BAX499/ARC19499	TFPI	Phase I [34, 35]
DNA aptamers	Thrombin	Research [11]
DNA aptamers	Phosphatidylserine (PS)	Research [11, 38] (see Section 2.4)
